# Studies to Assess the Utility of Serum Neurofilament Light Chain as a Biomarker in Chemotherapy-Induced Peripheral Neuropathy

**DOI:** 10.3390/cancers15174216

**Published:** 2023-08-23

**Authors:** Guido Cavaletti, Chiara Pizzamiglio, Albert Man, Thomas M. Engber, Cristoforo Comi, Darren Wilbraham

**Affiliations:** 1 Experimental Neurology Unit, School of Medicine and Surgery, University of Milano-Bicocca, 20900 Monza, Italy; 2 Fondazione IRCCS San Gerardo dei Tintori, 20900 Monza, Italy; 3 Department of Translational Medicine, University of Piemonte Orientale, 28100 Novara, Italy; 4 Department of Neuromuscular Diseases, UCL Queen Square Institute of Neurology, The National Hospital for Neurology and Neurosurgery, London WC1N 3BG, UK; 5 Eli Lilly and Company, Indianapolis, IN 46285, USAtengber@gmail.com (T.M.E.)

**Keywords:** chemotherapy-induced peripheral neuropathy, neurofilament light chain, paclitaxel, cancer, dose-limiting toxicity

## Abstract

**Simple Summary:**

Since neuroaxonal damage and loss are observed in chemotherapy-induced peripheral neuropathy (CIPN) and results in permanent disability, detecting and monitoring neuropathy with a serum biomarker would be advantageous in identifying the development, severity, and resolution of CIPN. We report here the results of two separate non-interventional studies (49 patients) that evaluated blood neurofilament light chain (NfL) as a biomarker of CIPN in breast cancer patients treated with paclitaxel. NfL was measured in serum using an ultrasensitive single-molecule array and compared with the self-administered European Organization for Research and Treatment of Cancer Quality of Life Questionnaire-CIPN twenty-item scale (CIPN20) and Total Neuropathy Score clinical version (TNSc), a clinician-reported measure of neuropathy progression. Both NfL and TNSc were associated with the cumulative dose of chemotherapy and CIPN20 sensory subscore after chemotherapy. These findings provided evidence that serum NfL in breast cancer patients treated with chemotherapy has the potential to be used as a biomarker to monitor and mitigate CIPN, although studies with additional patients planned in the ongoing clinical trial will determine the universal application of NfL as a biomarker in CIPN.

**Abstract:**

Chemotherapy-induced peripheral neuropathy (CIPN) is one of the most common and disabling dose-limiting toxicities of chemotherapy. We report here the results of two separate non-interventional studies (49 patients), which evaluated blood neurofilament light chain (NfL) as a biomarker of CIPN in breast cancer patients treated with paclitaxel. All patients underwent a standard treatment protocol that was established independently of the present studies. NfL was measured in serum using an ultrasensitive single-molecule array and compared with the self-administered European Organization for Research and Treatment of Cancer Quality of Life Questionnaire-CIPN twenty-item scale (CIPN20) and Total Neuropathy Score clinical version (TNSc), a clinician-reported measure of neuropathy progression. The TNSc increased with cumulative dose compared with baseline, and the NfL concentrations were also strongly associated with the cumulative dose of chemotherapy. The analysis showed a correlation between TNSc and NfL. Both TNSc and NfL showed weak to moderate associations with CIPN20 subscores, with a better association for the CIPN20 sensory compared with motor and autonomic subscores. Data from the two studies provide evidence that serum NfL has the potential to be used as a biomarker to monitor and mitigate CIPN. However, studies with additional patients planned in the ongoing clinical trial will determine the universal application of NfL as a biomarker in CIPN.

## 1. Introduction

Chemotherapy-induced peripheral neuropathy (CIPN) is a serious complication in cancer patients, as it can lead to a decline in quality of life and decreased patient survival [1,2]. CIPN occurs when peripheral nerves are damaged after exposure to neurotoxic chemotherapeutic agents such as taxanes, platins, and vinca alkaloid-derived chemotherapeutic agents. CIPN is one of the most common dose-limiting toxicities of chemotherapy [3], with symptoms including early post–treatment pain, paresthesia, sensory ataxia, and mechanical and cold allodynia. In early–stage breast cancer patients undergoing taxane-based chemotherapy in adjuvant and neoadjuvant settings, 17% of CIPN-associated dose reduction was reported [4], and such dose reductions can affect survival [5,6].

More than 60% of all patients treated with neurotoxic chemotherapy for cancer are affected by CIPN [7]. Approximately 70% of patients using paclitaxel [8], and 31% to 64% of patients treated with vinca alkaloids and proteasome inhibitors for hematological malignancies, reported CIPN symptoms [2]. Between 21% to 63% of patients using platin–based regimens for colorectal cancers and 63% of patients using thalidomide reported CIPN [8].

CIPN is dose–dependent, occurring as early as 24 h to 72 h following drug administration but often as late as 3 months after completion of chemotherapy, a phenomenon known as coasting. In many cases, it persists after discontinuation of treatment. Symptoms persist in 58% to 64% of breast cancer patients treated with taxanes who finished chemotherapy within 5 years [1]. The CIPN severity ranges from mild to severe, dependent on cumulative chemotherapy dose, history of neuropathy, and genetic polymorphisms [2].

Neuropathic symptoms such as sensory loss, paresthesia, dysesthesia, and numbness typically appear within 6 months of chemotherapy initiation [9,10]. The acute symptoms of CIPN often result in dose reduction and cessation of chemotherapy, which may impact overall cancer survival [5,6].

The reported incidence of CIPN has a wide range of variability, in part due to the lack of precision in clinical diagnosis. In practice, diagnosis is generally based on clinical assessment by the oncologist. Rarely, nerve conduction velocity, skin biopsy, or nerve biopsies are used [11]. Validated patient-reported outcome (PRO) and clinician-reported outcome (ClinRO) measures have been developed [12]. ClinRO scales used for CIPN grading include the physician-assessed Common Toxicity Criteria of the National Cancer Institute–Common Terminology Criteria for Adverse Events (NCI-CTCAE) and the Total Neuropathy Score clinical version (TNSc) [13,14]. PRO measures include the European Organization for Research and Treatment of Cancer Quality of Life Questionnaire-CIPN twenty-item scale (CIPN20) [15,16,17].

Although no specific mechanisms of platinum-based drug-induced CIPN have been clearly established, several factors such as activation of glia cells causing elevation of pro-inflammatory cytokines, mitochondria damage leading to ROS production, apoptosis, and hyperexcitability of peripheral neurons may be involved in CIPN [18]. Damage to central and peripheral neurons by these factors may cause the release of neurofilament light chain (NfL) into the cerebrospinal fluid (CSF) and blood. Since neuroaxonal damage and loss are observed in CIPN and can result in permanent disability, detecting and monitoring CIPN with a serum biomarker would be advantageous in identifying the development, severity, and resolution of CIPN. NfL, a neuronal cytoskeletal protein, is a promising biomarker in neurodegenerative disorders and has gained significant attention as a serum biomarker of axonal degeneration. Abnormal levels of NfL in the CSF and blood reflect axonal damage in a variety of neurodegenerative, inflammatory, vascular, and traumatic conditions. Studies have demonstrated that NfL levels are increased in a wide range of neurologic disorders, including multiple sclerosis, amyotrophic lateral sclerosis (ALS), traumatic brain injury, and Parkinson’s disease [19,20,21,22]. Recent studies have also demonstrated the utility of NfL as a biomarker of neuroaxonal damage in patients treated with oxaliplatin and paclitaxel [23,24]. The purpose of this report is to determine the utility of NfL as a biomarker of CIPN in breast cancer patients receiving paclitaxel. Data from these studies provide evidence that serum NfL has the potential to be used as a biomarker for the development of CIPN.

## 2. Materials and Methods

### 2.1. Patients

Patients who participated in the Epiphany trial (NCT03997981) and the investigator-initiated trial (Novara study; protocol number 128/13 approved by the local Ethics Committee) were included in this analysis. Epiphany is an ongoing prospective natural history study of CIPN in patients receiving taxanes (paclitaxel, docetaxel) for breast cancer, bortezomib for multiple myeloma, oxaliplatin-based regimens for colorectal cancer, or vincristine for lymphoma. This report includes data from breast cancer patients receiving weekly paclitaxel prior to a recruitment pause due to SARS-CoV-2 (COVID-19). This study is a multi-site study in the US. The investigator-initiated Novara study was a longitudinal, observational study in patients with a diagnosis of breast cancer referred to the Department of Translational Medicine (University Hospital, Novara, Italy) between January 2017 and January 2019 [25].

### 2.2. Standard Protocol Approvals, Registrations, and Patient Consents

The ethics committees of all participating centers approved the protocol, and the trial was conducted in accordance with the Declaration of Helsinki. All patients included in these studies signed an informed consent prior to joining the study.

### 2.3. Study Design

Novara Study: All patients had to meet the following criteria: (1) ≥18 years of age, (2) Karnofsky performance status index > 70, and (3) breast cancer requiring chemotherapy (N0 or N1) without systemic metastasis. Patients with prior neurotoxic chemotherapy were excluded from participation.

All patients in the Novara study underwent a standard treatment protocol that was established independently of the present study. The treatment for the patients included in this analysis consisted of neoadjuvant chemotherapy with anthracyclines (fluorouracil, epirubicin, and cyclophosphamide followed by taxanes [paclitaxel 80 mg/m^2^ weekly for 12 weeks]). Patients were evaluated at three different time points: at baseline (before taxane [T0]), 1 month (T1), and 3 months (T2) after the beginning of taxane. Demographic and clinical data were collected from oncology records.

The clinical evaluation was focused on the neuropathy assessment. A complete neurological examination was performed at each visit (T0, T1, and T2), including assessment of sensory (pain, temperature, and vibration), motor (Medical Research Council grade 5 score for muscle strength), and deep tendon reflexes. At each visit, neuropathy progression was quantified with the Total Neuropathy Score-reduced (TNSr) [13], and the TNSc scores for the Novara study presented in this report were calculated post hoc.

Quality of life was evaluated at each visit with the self-administered CIPN20 [15]. This questionnaire, which specifically evaluates neuropathic symptoms and their impact on daily life, comprises three sections: sensory (nine items), motor (eight items), and autonomic (two items). Raw and standardized scores were calculated for each section. Only standardized (0–100) scores are reported in this article. Nerve conduction studies were performed but are not presented in this report.

Epiphany Study: All patients have to meet the following criteria: (1) ≥18 years, (2) life expectancy ≥ 6 months, (3) patients with NCI-CTCAE grade 0 CIPN (with the exception of patients with multiple myeloma treated with bortezomib, NCI-CTCAE grade ≤ 1), (4) Eastern Cooperative Oncology Group performance status of 0–2, and patients with (5a) breast cancer beginning treatment with paclitaxel or docetaxel with curative intent, with a minimum of six cycles of chemotherapy planned, (5b) lymphoma initiating treatment with vincristine, with a minimum of four cycles of chemotherapy planned, (5c) Stage III (may consider Stage IV with minimal metastasis) colorectal cancer initiating oxaliplatin-based regimens, a total of 6 months, with a minimum of 12 cycles planned, or (5d) multiple myeloma initiating bortezomib, a total of 4 months, with a minimum of nine cycles planned. Enrollment must be completed prior to receiving the first dose of neurotoxic chemotherapy. Data from breast cancer patients treated with weekly paclitaxel are presented in this report. Patients receiving combination treatment with multiple neurotoxic agents or other single neurotoxic agents are not presented in this report, as the aim of this report was to investigate the relationship of biomarkers such as NfL in patients receiving paclitaxel as the primary neurotoxic chemotherapy agent.

During the study, patients were evaluated for the development of CIPN using electronic PROs and ClinROs. ClinRO scales used for CIPN grading included the physician-assessed common toxicity criteria of the NCI-CTCAE and the TNSc [13,14]. PRO measures included the CIPN20 [15,16,17].

### 2.4. Biomarker

Blood samples (5 mL) were collected from the patients at baseline, each visit during the observational period, and up to 6 months after the last dose of chemotherapy. Blood was processed, and serum was stored according to standard procedures. The serum was assayed in duplicates using the ultrasensitive single-molecule array (Simoa^®^ NF-Light™ assay, Quanterix, Lexington, MA, USA) to determine NfL concentration. Additional details, including the lower limit of quantification and the limit of detection, can be found in the Simoa^®^ NF-Light™ Advantage Kit HD-1/HD-X Data Sheet. The intra-assay and inter-assay coefficient of variations were less than 3.6%.

### 2.5. Statistical Methods

Descriptive summary statistics are provided for patient demographics and disease characteristics. Repeated measures correlation was used to assess the correlation between two variables measured over time [26,27]. *T*-tests were used to assess differences between variables at two time points or between two different groups. Natural log transformations were applied to normalize variables where appropriate, such as NfL. Only NfL was natural log transformed to normalize, as done in other studies [28,29]. Statistical significance was assessed at the α = 0.05 level, and *p*-values were not adjusted for multiplicity. All programming was conducted in version 3.6.2 of the R language for statistical computation and graphics from the R Foundation [30].

## 3. Results

### 3.1. Patients

Baseline characteristics of the patients are described in Table 1.

Female patients with breast cancer treated with paclitaxel were included in this analysis. All 30 patients enrolled in the Novara study who were treated with paclitaxel were included in this report. Among the 50 patients enrolled in the Epiphany study, only those treated with paclitaxel (19 patients) were included. The mean (standard deviation) age of the patients was 54.0 (9.6) years in the Novara study and 52.8 (12.4) years in the Epiphany study. Most patients had Stage II disease at diagnosis in the Novara study, and disease stage was not reported in the Epiphany study. Fourteen (46.7%) patients in the Novara study had a paclitaxel dose reduction due to side effects (neuropathic pain in five patients, gastrointestinal toxicity in three patients, hematologic toxicity in one patient, liver toxicity in two patients, liver toxicity plus hematologic toxicity in one patient, pneumonia in one patient, and unknown cause in one patient). In the Epiphany study, seven (36.8%) patients had dose reductions, with no reasons for dose reduction reported in these patients. In the Epiphany study, the decision to reduce the dose was made at the discretion of the treating physician in line with local practice. No patients had signs of peripheral neuropathy, as determined by TNS, at baseline. For those patients whose paclitaxel dose was reduced, the decision was made by the oncologist during the weekly follow-up appointments.

### 3.2. TNSc, NfL, and CIPN20 Sensory, Motor, and Autonomic Subscore Changes during Chemotherapy in Novara and Epiphany Studies

**Figure 1:**
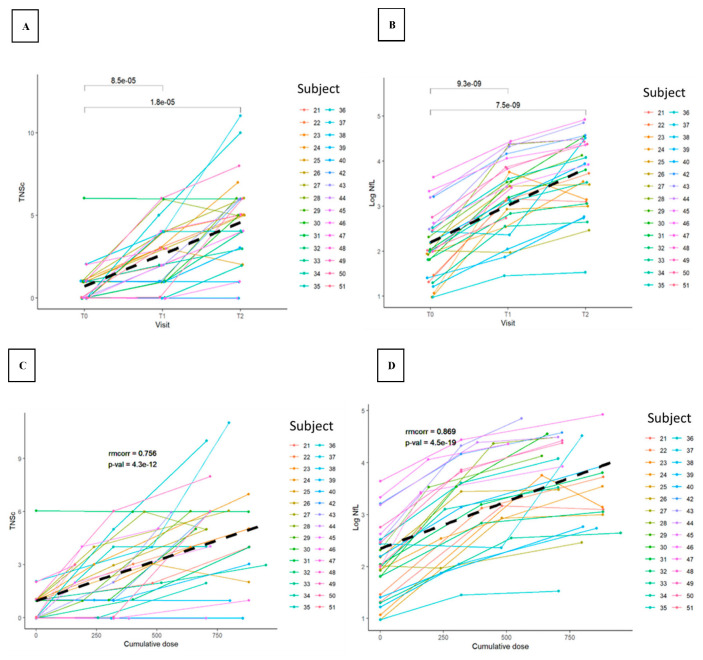
TNSc (**A**,**C**) and log NfL (**B**,**D**) changes versus visit (**A**,**B**) and cumulative dose (**C**,**D**) during chemotherapy in the Novara study. Patients 25, 26, 27, 28, 29, 30, 31, 34, 35, 36, 43, 44, 46, and 50 had a paclitaxel dose reduction. Abbreviations: NfL = neurofilament light chain; *p*-val = *p*-value; rmcorr = repeated measures correlation coefficient; T0 = before taxane; T1 = 1 month after taxane; T2 = 3 months after taxane; TNSc = Total Neuropathy Score clinical version. Note: In the top-row figures, the annotations in the plot are unadjusted *p*-values (*t*-tests) for the differences between visits or cycles.

**Figure 2:**
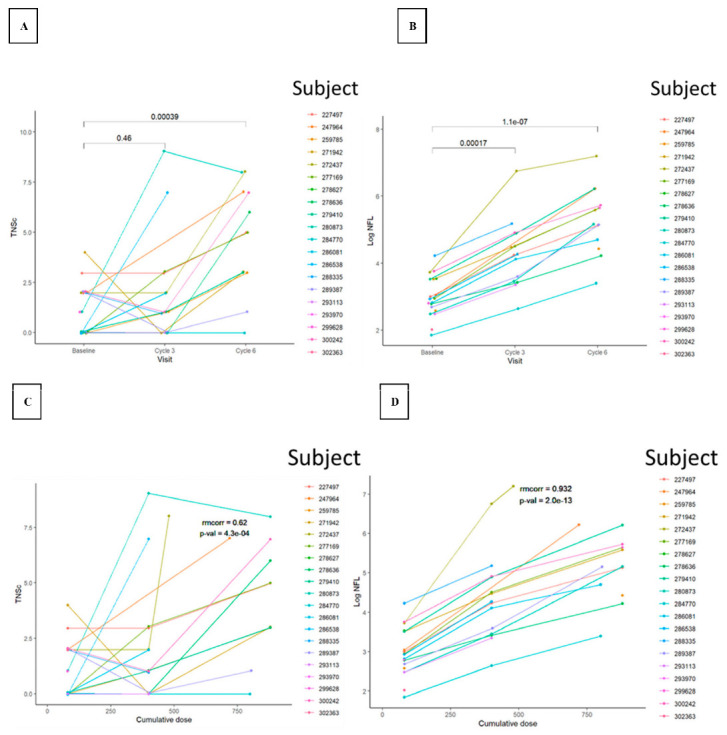
TNSc (**A**,**C**) and log NfL (**B**,**D**) changes versus visit (**A**,**B**) and cumulative dose (**C**,**D**) during chemotherapy in the Epiphany study. Patients 247964, 272437, 278627, 284770, 286081, and 289387, 302363 had a paclitaxel dose reduction. Abbreviations: log_NfL = log neurofilament light chain; *p*-val = *p*-value; rmcorr = repeated measures correlation coefficient; TNSc = Total Neuropathy Score clinical version. Note: In the top-row figures, the annotations in the plot are unadjusted *p*-values (*t*-tests) for the differences between visits or cycles.

The baseline levels of TNSc ranged from zero to approximately six (Figure 1 and Figure 2).

**Figure A1:**
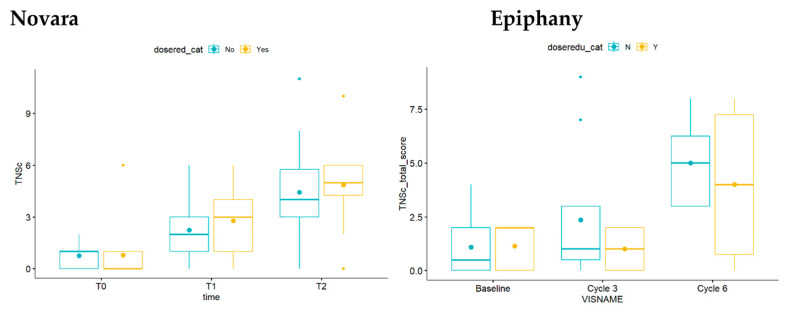
The effect of dose reduction on TNSc in the Novara and Epiphany studies. Fourteen patients (Patient IDs: 25, 26, 27, 28, 29, 30, 31, 34, 35, 36, 43, 44, 46, and 50) in Novara and seven patients (Patient IDs: 247964, 272437, 278627, 284770, 286081, 289387, and 302363) in Epiphany had dose reductions. Abbreviations: doseredu_cat = dose reduction category; N = no; T0 = before taxane; T1 = 1 month after taxane; T2 = 3 months after taxane; TNSc = Total Neuropathy Score clinical version; VISNAME = visit name; Y = yes.

**Figure A2:**
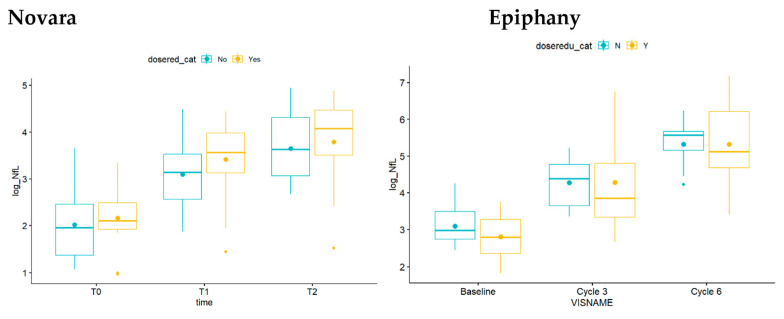
The effect of dose reduction on NfL in the Novara and Epiphany studies. Fourteen patients (Patient IDs: 25, 26, 27, 28, 29, 30, 31, 34, 35, 36, 43, 44, 46, and 50) in Novara and seven patients (Patient IDs: 247964, 272437, 278627, 284770, 286081, 289387, and 302363) in Epiphany had dose reductions. Abbreviations: doseredu_cat = dose reduction category; N = no; NfL = neurofilament light chain; T0 = before taxane; T1 = 1 month after taxane; T2 = 3 months after taxane; VISNAME = visit name; Y = yes.

**Figure A3:**
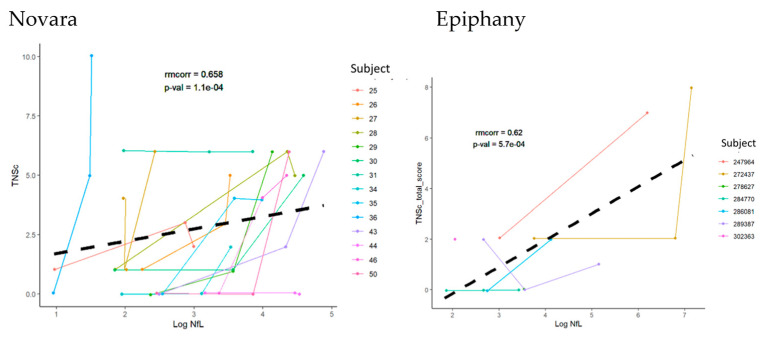
The effect of dose reduction on TNSc versus NfL in the Novara and Epiphany studies. Fourteen patients (Patient IDs: 25, 26, 27, 28, 29, 30, 31, 34, 35, 36, 43, 44, 46, and 50) in Novara and seven patients (Patient IDs: 247964, 272437, 278627, 284770, 286081, 289387, and 302363) in Epiphany had dose reductions. Abbreviations: NfL = neurofilament light chain; *p*-val = *p*-value; rmcorr = repeated measures correlation coefficient; TNSc = Total Neuropathy Score clinical version; VISNAME = visit name.

The TNSc increased with cumulative dose compared with baseline, with a repeated measures correlation coefficient (rmcorr) of 0.756 (*p* = 4.3 × 10^−12^) and 0.620 (*p* = 4.3 × 10^−4^) for Novara (Figure 1) and Epiphany (Figure 2), respectively. A similar trend was also observed for T2 (Novara; *p* = 1.8 × 10^−5^) and cycle six (Epiphany; *p* = 0.00039) compared with baseline. Several patients had high baseline NfL concentrations, reaching a maximum of 70.0 pg/mL in the Epiphany study and a maximum of 38.6 pg/mL in the Novara study. NfL concentrations were also strongly associated with the cumulative dose of chemotherapy, with an rmcorr of 0.869 (*p* = 4.5 × 10^−19^) and 0.932 (*p* = 2.0 × 10^−13^) for Novara and Epiphany, respectively, and a similar trend observed for visits (T2 [Novara: *p* = 7.5 × 10^−9^] and cycle six [Epiphany: *p* = 1.1 × 10^−7^]) compared with baseline. No measurable difference was observed between patients with a dose reduction and patients with no dose reduction in terms of TNSc (Figure A1) and NfL (Figure A2), or in the relationship between NfL and TNSc (Figure A3).

**Figure 3:**
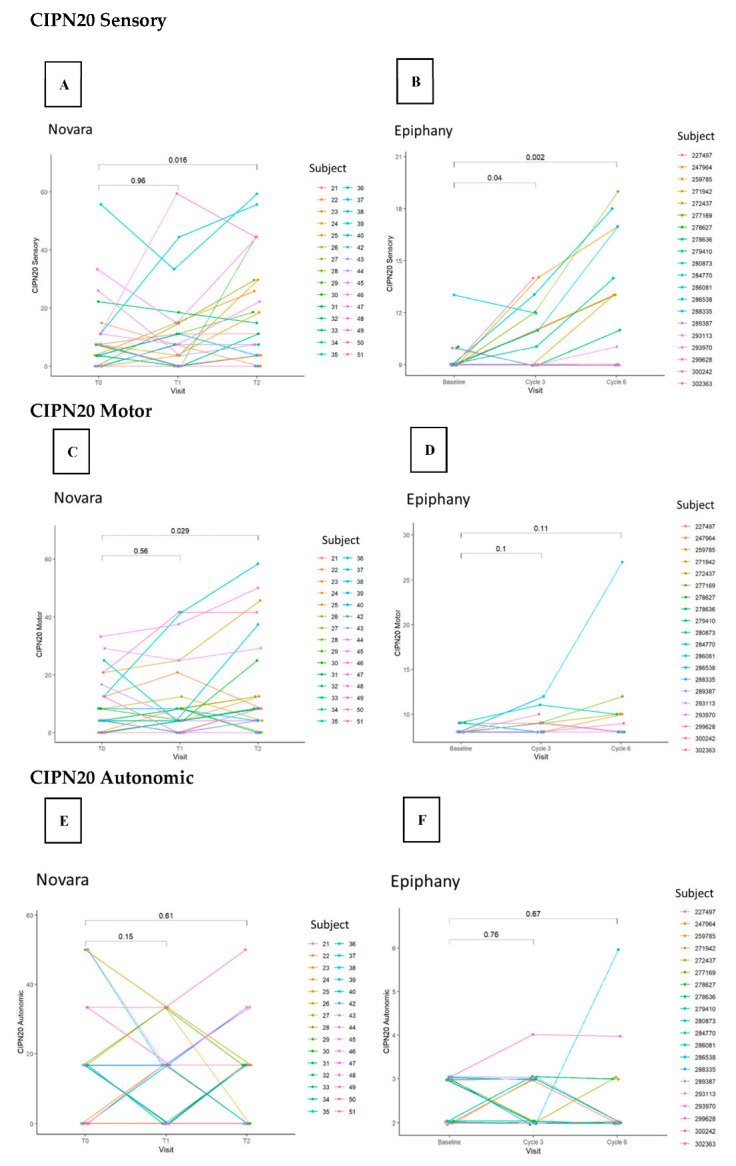
CIPN20 sensory (**A**,**B**), motor (**C**,**D**), and autonomic (**E**,**F**) correlation during chemotherapy in the Novara and Epiphany studies. Abbreviations: CIPN20 = Chemotherapy-Induced Peripheral Neuropathy twenty-item scale; T0 = before taxane; T1 = 1 month after taxane; T2 = 3 months after taxane. Note: The annotations in the plot are unadjusted *p*-values (*t*-tests) for the differences between visits or cycles.

Changes from baseline to final visits were observed in sensory symptoms (Novara: *p* = 0.016; Epiphany: *p* = 0.002), inconclusive in motor symptoms (Novara: *p* = 0.029; Epiphany: *p* = 0.11), and not different in autonomic symptoms (Novara: *p* = 0.61; Epiphany: *p* = 0.67) (Figure 3).

### 3.3. Repeated Measures Correlation between TNSc, NfL, and CIPN20 Sensory, Motor, and Autonomic Changes in Novara and Epiphany Studies

**Figure 4:**
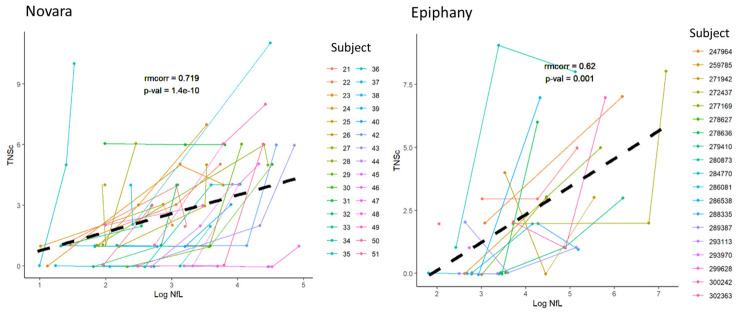
TNSc correlation with NfL in the Novara (**A**) and Epiphany (**B**) studies. Abbreviations: NfL = neurofilament light chain; *p*-val = *p*-value; rmcorr = repeated measures correlation coefficient; TNSc = Total Neuropathy Score clinical version.

Repeated measures correlation analysis showed a good correlation between TNSc and NfL, with an rmcorr of 0.719 (*p* = 1.4 × 10^−10^) and 0.62 (*p* = 0.001) for Novara and Epiphany, respectively (Figure 4).

**Figure 5:**
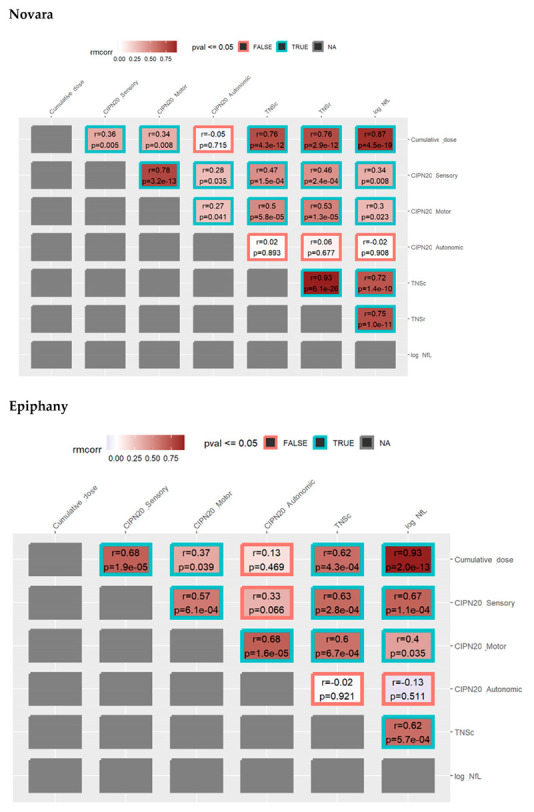
TNSc repeated measures correlation with cumulative dose, CIPN20 sensory, motor, and autonomic, TNSr (not measured in Epiphany), and log NfL in the Novara and Epiphany studies. Epiphany only assessed TNSc, and therefore, TNSr could not be calculated. Abbreviations: CIPN20 = Chemotherapy-Induced Peripheral Neuropathy twenty-item scale; NfL = neurofilament light chain; NA = not available; *p*/*p*val = *p*-value; r/rmcorr = repeated measures correlation coefficient; TNSc = Total Neuropathy Score clinical version; TNSr = Total Neuropathy Score-reduced.

The rmcorr for the CIPN20 subscores with cumulative dose were similar between the two studies, with sensory subscores (Novara: 0.36 [*p* = 0.005]; Epiphany: 0.68 [*p* = 1.9 × 10^−5^]) exhibiting a better association than the motor (Novara: 0.34 [*p* = 0.008]; Epiphany: 0.37 [*p* = 0.039]) and autonomic (Novara: −0.05 [*p* = 0.715]; Epiphany: 0.13 [*p* = 0.469]) subscores (Figure 5).

The CIPN20 sensory and motor subscores showed moderate associations with TNSc (range: 0.47–0.63) in both studies. The CIPN20 motor subscores showed weak associations with NfL (0.30 and 0.40) in both studies. However, the CIPN20 sensory subscore showed a moderate correlation with NfL (0.67) in the Epiphany study, and a weak association was observed in the Novara study (0.34). The CIPN20 autonomic subscore had the weakest correlation with TNSc and NfL (−0.13 to 0.02), and it displayed the least change from baseline. In the Novara study, the correlation coefficient between TNSc and TNSr was 0.93, with similar correlation coefficients between TNSc and TNSr for cumulative dose and CIPN20 sensory, motor, and autonomic subscores.

## 4. Discussion

The lack of effective evidence–based treatments for CIPN highlights the need for high-quality clinical trials to identify interventions that can prevent or treat this condition. Ongoing clinical programs include driving pharmacological research toward the prevention and treatment of CIPN, which are developing drug regimen strategies to minimize CIPN occurrence [30,31,32,33]. Neurofilaments have gained significant attention as biomarkers of axonal injury [34]. These abundant structural scaffolding proteins are exclusively expressed in neurons [20] and are thought to be critical for radial growth and the stability of axons, enabling effective nerve conduction [35]. Neurofilaments are specific indicators of axonal injury and thus offer significant advantages over other biomarkers [36]. Abnormal levels of NfL in the CSF and blood reflect axonal damage in a variety of neurodegenerative, inflammatory, vascular, and traumatic conditions [37,38,39]. NfL levels increase in the early clinical phase of patients with ALS, and higher levels are associated with disability, relapse status, risk of future relapse, and worsening of disability in patients with multiple sclerosis [40].

The fourth-generation single molecular array (SiMoA) technology has allowed for reliable quantification of NfL levels in CSF, plasma, or serum across a range of concentrations observed in disease and physiological conditions [40]. The assay is highly sensitive and has a broad dynamic range, requiring a small sample size. The SiMoA assay has been used to demonstrate elevated blood levels of NfL in ALS, multiple sclerosis, oxaliplatin-induced peripheral neuropathy, and paclitaxel-induced neuropathy [19,20,23,41].

**Figure A4:**
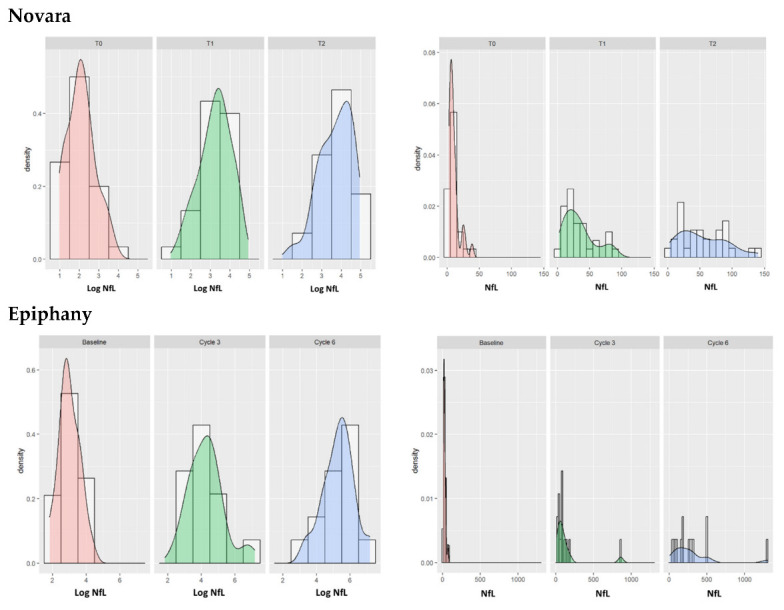
Distribution of log NfL and NfL at each time point. Abbreviations: NfL = neurofilament light chain; T0 = before taxane; T1 = 1 month after taxane; T2 = 3 months after taxane.

In this report, we compared serum NfL levels measured using SiMoA technology with the TNSc, as well as the CIPN20 sensory, motor, and autonomic measurements during paclitaxel treatment in patients taking part in the Novara and Epiphany studies. The patients included in these studies were representative of breast cancer patients treated with paclitaxel. Baseline characteristics of patients in the two trials were similar. The distributions of NfL and log NfL at each time point are shown in Figure A4. This study demonstrated that serum NfL levels and TNSc are closely related to cumulative dose in the two different studies. However, while a moderate correlation was observed between CIPN20 sensory measures and cumulative dose, autonomic and motor measures showed only a weak association.

The comparison among the measures (cumulative dose, CIPN20 [sensory, motor, and autonomic], TNSc, and NfL) in these patients showed a moderate to strong correlation between NfL and TNSc in both studies. The CIPN20 sensory subscore correlated well with NfL in Epiphany and poorly in Novara. This is consistent with previous studies that showed the CIPN20-specific submodule is a valid tool in sensory CIPN detection [15,42,43,44], suggesting that paclitaxel-induced neuropathy is primarily sensory in nature. However, a recent study also showed a good correlation between NfL and CIPN20 in the progression of oxaliplatin-induced peripheral neuropathy [23]. The results of our studies are consistent with a recent report that showed a correlation between NfL levels and paclitaxel-induced peripheral neuropathy at the end of treatment [41]. Another recent study demonstrated a correlation between serum NfL and increase in CIPN that could be used to identify subjects susceptible to dose-limiting paclitaxel and carboplatin-induced CIPN before the onset of symptoms [45]. A meta-analysis that included 36 studies reporting on 4414 participants, including 2301 patients with peripheral neuropathy and 2113 controls, showed that NfL was significantly increased in patients with peripheral neuropathy compared with controls [46]. Unlike the published report that compared NfL with TNSc only, our results also included PROs that indicated paclitaxel-induced peripheral neuropathy is primarily sensory in nature. Overall, our current study together with the recent published reports [15,23,41,42,43,44,46] support the potential use of NfL as a biomarker in CIPN.

The current report has some strengths, including the evaluation of a homogenous breast cancer population receiving a paclitaxel regimen and data collection from multiple sites (Epiphany), although Novara was a single–site study. However, both studies had small sample sizes; a larger sample size is needed to determine if the patients’ responses to the CIPN20 questionnaire were biased due to patients’ motivation to remain in the study and the impact of such bias on the CIPN20 evaluations. Furthermore, the correlation between TNSc and NfL is lower in Epiphany compared with Novara, which is likely due to conduct across multiple sites with less experience in using the TNS scale, as reflected in the greater fluctuation of TNS data in the Epiphany study. Epiphany recruitment activities have recently restarted, with a plan for data collection from up to 200 patients by staff with better training in using the TNSc, which will add valuable additional data for the analysis presented in this paper. Breast cancer is a heterogenous disease with various molecular subtypes, and thus different treatment options are available to patients. Future studies with a larger population of breast cancer patients receiving combination treatment with multiple neurotoxic agents or other single neurotoxic agents could determine the potential use of NfL as a biomarker in CIPN. Overall, the data from these two studies indicate that serum NfL has the potential to be used as a biomarker in CIPN to monitor and mitigate the CIPN, although studies with additional patients planned in the ongoing clinical trial will determine the universal application of NfL as a biomarker in CIPN.

## 5. Conclusions

NfL has gained significant attention as a serum biomarker of neuroaxonal damage in patients treated with chemotherapy. Our study analyzed the results of two separate non-interventional studies to determine the utility of NfL as a biomarker of CIPN in breast cancer patients receiving paclitaxel. All patients underwent a standard treatment protocol, and the NfL levels were compared with the TNSc, as well as CIPN20 sensory, motor, and autonomic measurements. The results showed a moderate to strong correlation between NfL and TNSc in both studies. The CIPN20 sensory subscore also correlated well with NfL. In conclusion, these non-interventional studies in breast cancer patients treated with chemotherapy provide evidence that serum NfL has the potential to be used as a biomarker to monitor and mitigate CIPN, although studies with additional patients planned in the ongoing clinical trial will determine the universal application of NfL as a biomarker in CIPN.

## Figures and Tables

**Table 1:** Overview of patient and tumor characteristics as well as chemotherapy treatment.

	Epiphany	Novara
Total, n	19	30
Sex, female (%)	19 (100.0)	30 (100.0)
Age, years (SD)	52.8 (12.4)	54.0 (9.6)
Cancer stage, n (%)		
I	NR	10 (33.3)
II	NR	14 (46.7)
III	NR	6 (20.0)
IV	NR	0 (0)
Dose reduction, n (%)	7 (36.8)	14 (46.7)
Neuropathic pain, n	NR	6
Hematologic toxicity, n	NR	4
PAC cumulative dose, mg/m^2^ (SD)		
T1	370.7 (81.0)	332.1 (115.7)
T2	835.6 (103.7)	771.5 (111.1)
BMI, average (SD)	31.6 (7.8)	22.3 (2.0)

Abbreviations: BMI = body mass index; n = number of patients; NR = not reported; PAC = paclitaxel; SD = standard deviation; T1 = 1 month after taxane; T2 = 3 months after taxane.

## Data Availability

All preliminary data analyzed are included in this report. The anonymized data are not publicly available due to privacy restrictions and will be made available upon request from any qualified investigator. Eli Lilly and Company’s policy is to provide access to all individual participant data collected during the trial, after anonymization, with the exception of pharmacokinetic or genetic data. No expiration date of data requests is currently set once data are made available. Access is provided after a proposal has been approved by an independent review committee identified for this purpose and after receipt of a signed data sharing agreement. Data and documents, including the study protocol, statistical analysis plan, clinical study report, and blank or annotated case report forms, will be provided in a secure data sharing environment. For details on submitting a request, see the instructions provided at www.vivli.org.
